# Evaluation of a Multifunctional Polyvinylpyrrolidone/Hyaluronic Acid-Based Bilayer Film Patch with Anti-Inflammatory Properties as an Enhancer of the Wound Healing Process

**DOI:** 10.3390/pharmaceutics14030483

**Published:** 2022-02-22

**Authors:** Marco Contardi, Maria Summa, Pasquale Picone, Ornella Roberta Brancato, Marta Di Carlo, Rosalia Bertorelli, Athanassia Athanassiou

**Affiliations:** 1 Smart Materials, Istituto Italiano di Tecnologia, Via Morego 30, 16163 Genova, Italy; 2 Translational Pharmacology, Istituto Italiano di Tecnologia, Via Morego 30, 16163 Genova, Italy; maria.summa@iit.it (M.S.); rosalia.bertorelli@iit.it (R.B.); 3 Istituto per la Ricerca e l’Innovazione Biomedica (IRIB), CNR, Via Ugo la Malfa 153, 90146 Palermo, Italy; pasquale.picone@irib.cnr.it (P.P.); ornellabran@virgilio.it (O.R.B.); marta.dicarlo@irib.cnr.it (M.D.C.)

**Keywords:** wound dressings, chronic wounds, polyvinylpyrrolidone, hyaluronic acid, anti-inflammatory, hemocompatibility, wound healing

## Abstract

The management of acute and chronic wounds is still a socioeconomic burden for society due to the lack of suitable tools capable of supporting all the healing phases. The exponential spread of diabetes worldwide and the consequent increase of complicated diabetic ulcers require further efforts to develop scalable, low-cost, and easy-to-use treatments for tackling this emergency. Recently, we explored the fabrication of a polyvinylpyrrolidone/hyaluronic acid-based bilayer wound dressing, characterizing its physicochemical features and detailing its excellent antimicrobial activity. Here, we further demonstrate its biocompatibility on fibroblasts, keratinocytes, and red blood cells. The bilayer shows anti-inflammatory properties, statistically reducing the level of IL-6, IL-1β, and TNF-α, and a capacity to accelerate wound healing in vitro and in healthy and diabetic mice models compared to untreated mice. The outcomes suggest that this bilayer material can be an effective tool for managing different skin injuries.

## 1. Introduction

Wound repair is considered one of the most complex biological procedures of our body, and it is divided into four main phases: hemostasis, inflammation, proliferation, and remodelling [1]. In normal healing, these phases occur in a sequential but overlapped way, and they are completed in a period between three weeks and some months, leading to the formation of scar tissue [2,3]. Unfortunately, various complications can occur and affect the damage recovery time required. For instance, bacteria such as *Pseudomonas aeruginosa* and *Staphylococcus aureus* can readily colonize wounds, causing aggressive and intricate infections [4,5]. Due to the release of proteolytic enzymes in the wound bed, these bacterial strains can therewithal decelerate the immune system response and digest the collagen that is the most important extracellular protein in granulation tissue [6,7]. Moreover, age-related immune deficiency, hypoxia, immobility, and pathological conditions such as hyperglycemia due to diabetes and obesity [8,9] can affect the success of healing. Diabetes represents a good model of a compromised wound closure process because several factors contribute to the healing failure. An altered ability to modulate the release of cytokines, important mediators that perform multiple functions in the inflammatory phase, and a high level of matrix metallo-proteinases lead to an arrest at the very early stages of the wound repair process [10]. Diabetic vascular trouble could reduce oxygen transport to the wound site and the function of red blood cells of carrying nutrients to the tissues, which in turn reduces the efficiency of the white blood cells to contrast eventual microorganism infections [11]. In addition, the re-epithelialisation phase, in which the different skin layers regenerate from the border to the center of the lesion, may be prevented by the lack of essential growth factors directly involved in this process [12,13]. These conditions can steer the repairing process to a prolonged impairment defined as chronic wounds, such as ulcerative lower limb lesions, which are dangerous for the patients and expensive in terms of drug consumption and medical assistance [3,9,14,15,16].

Current commercial “passive” dressings such as cotton, bandages, and gauzes are used as first aid tools in the case of burns and wounds to cover and protect the injury from contaminations and excessive loss of fluids [17]. However, these tools tend to fail when the aforementioned severe complications arise, requiring the use of specific and effective therapies.

Advanced dressings can deliver bioactive molecules such as antibiotics [18], antioxidants [19,20], anti-inflammatory [21,22], and growth factors [23] able to better sustain the healing process [24,25,26,27]. Both synthetic and natural polymers have been used for the fabrication of functional and biocompatible dressings in the form of hydrogels, electrospun nanofibers, thin films, and 3D printed biomaterials [17,28,29,30,31]. These formulations can ensure good oxygenation, suitable absorption of exudates, and support during the proliferation phase [27,28,32].

Additionally, therapeutic dressings should be well-tolerated by the skin without triggering any reaction while being in contact with the tissue—healthy or damaged—and with the body fluids [33,34]. Furthermore, proper and responsive adhesion and mechanical ductility are required to provide the patients with an easily-applied and comfortable bandage [35,36].

Recently, Heyer et al. [37] described how the introduction of the aforementioned dressings has led to an enhancement of 52% of the rate of the repairing process compared to traditional dressings in chronic wound cases. On the other hand, Derakhshandeh et al. [38] highlighted some limitations of the advanced dressings, such as the inability to see or understand the state of the wound, the necessity of constant medical assistance, and management of the therapy during the different phases of the healing process. Furthermore, the production cost of these advanced tools calls for innovative, scalable, and eco-friendly methods of fabrication [39,40]. Therefore, the design of an ideal material/formulation including all the mentioned requirements is still a great challenge for researchers.

In a recent study [41], we presented the fabrication of a PVP-based multifunctional bilayer material using two water-based scalable approaches. The bilayer construct was composed of polyvinylpyrrolidone and hyaluronic acid and was loaded with an antiseptic, Neomercurocromo^®^, and an antibiotic, ciprofloxacin. The material allowed the monitoring of the state of the wound due to its transparency, showing responsive and tunable adhesion to the skin in dry and wet conditions, and sequential delivery of the two loaded drugs. These features are connected with the PVP capacity to produce transparent film materials [18] that are adhesive, and the interactions between PVP and hyaluronic acid in the matrix modulate the release of the two drugs. Moreover, it exhibited efficient activity against three bacterial strains, *Escherichia coli*, *Staphylococcus aureus*, and *Pseudomonas aeruginosa*, and displayed suitable biocompatibility and complete resorption by the wound in an in vivo mice model.

Here, we aim to explore the multifunctionality of the bilayer construct further, evaluating its effect in several in vitro and in vivo mice models.

## 2. Materials and Methods

### 2.1. Materials

Polyvinylpyrrolidone (PVP; MW = 360,000), monohydrochloride monohydrate free ciprofloxacin (Cipro; ≥98.0% HPLC; MW = 331.34), glycerol, acetic acid (AcOH; ≥99.7%), and phosphate buffered saline (PBS) solution (pH 7.4) were bought from Sigma-Aldrich (St. Louis, MO, USA). Hyaluronic acid sodium salt (HA; MW = 350,000) was bought from Abcr (Karlsruhe, Germany). All the purchased products were used as received. A Milli-Q Advantage A10 ultrapure water purification system was utilized for the deionized water. Neomercurocromo^®®^ (Neo), a commercialized cutaneous antiseptic product, produced by Laboratorio Farmaceutiche Specialità Igienico Terapeutiche S.I.T. s.r.l. (Pavia, Italy), was bought in a local pharmacy. Further details regarding Neo composition and its application can be found in Contardi et al. [41].

### 2.2. Bilayer Preparation

The bilayer wound dressing films were fabricated by following the procedure reported in Contardi et al. [41]. In summary, the first layer was prepared by solvent casting method starting from an acetic acid (AcOH)/water solution (1% *v/v* in AcOH) made of hyaluronic acid 2% (*w*/*v*), polyvinylpyrrolidone 2% (*w*/*v*), ciprofloxacin 0.014% (*w*/*v*), and glycerol (to a concentration of 10 wt% compared to total polymer dry film weight). For the production of the second layer, a water-based solution composed of PVP 20% (*w*/*v*), glycerol (10 wt% dry basis), and Neomercurocromo^®®^ 10% (*v*/*v*) was spread on the first layer using two methodologies: spin-coating and rod-coating. In this work, we used the spin-coating method because we did not need large-size and highly numerous samples for the in vitro and in vivo tests that we performed. Therefore, samples of the first layer with a round shape (diameter of 5 cm) were prepared. Afterwards, the second layer was obtained using a Spin-coater (model WS-650S-6NPP/LITE/OND by Laurell Technologies Corporation, Philadelphia (North Wales), US) over the initial dry film. More details are presented in Contardi et al. [41].

### 2.3. Cell Culture and Treatments

HaCaT cell line were purchased from the Cell Line Service (Heidelberg, Germany). Cells were cultured in DMEM supplemented with 10% fetal bovine serum (FBS) and 2 mmol/L l-glutamine at 37 °C in an atmosphere of 5% CO_2_ and 95% air.

Primary human dermal fibroblasts (HDFa) (Thermo Fisher Scientific, Milan, Italy) were cultured with DMEM medium (Celbio, Milan, Italy) supplemented with 10% fetal bovine serum (FBS) (Gibco-Invitrogen, Milan, Italy), 2 mM glutamine, 1% penicillin, and 1% streptomycin (50 mg/mL). Cells were maintained in a humidified 5% CO_2_ atmosphere at 37 ± 0.1 °C.

### 2.4. Cytotoxicity Assay

The first in vitro cytotoxicity assessment was conducted on HaCaT cells using CellTiter-Glo Luminescent viability assay (Promega Italia S.r.l., Milan, Italy). In 96-well plates. HaCaT cells were placed at a density of 3.5 × 10^5^ in a final medium well volume of 100 μL and incubated until the proper confluence was reached. After 24 h of treatment, cells were quickly rinsed with pre-warmed PBS with Ca^2+^/Mg^2+^, and the extraction medium was substituted with the extraction one (control samples were treated with medium processed as the extractions). Afterwards, cells were incubated for an additional 24 h and 48 h. Extracts were produced by placing bilayer films in cell medium at different concentrations. According to ISO10993-5 guidelines, as the cell viability of the sample extracts were higher than 70% of the control group, all materials were considered biocompatible. Cell viability was determined by measuring ATP levels by CellTiter-Glo assay, as indicated by the supplier as percentage survival relative to control cells. Data represent mean ± SD of three independent experiments. The impact of bilayer films on cell morphology was also monitored using an LEICA DMI6000B inverted microscope.

For the cytocompatibility assay using HDFa, cells were seeded on 96-well flat-bottom plates at a density of 4 × 10^3^ per well. Different amounts (2.5, 5.0, 10.0 mg) of bilayer were added onto a HDFa monolayer, and the samples were incubated at 37 °C for 48 h. Cell viability was investigated by MTS assay (Promega Italia S.r.l., Milan, Italy). MTS [3-(4,5-dimethylthiazol-2-yl)-5-(3-carboxymethoxyphenyl)-2-(4-sulphophenyl)-2H-tetrazolium] was employed according to the manufacturer’s instructions. HDFa cells were placed in a 96-well plate and, after treatment, 20 μL of the MTS solution was placed into each well and incubated for 4 h at 37 °C in a humidified incubator with 5% CO_2_. A Microplate reader Wallac Victor 2 1420 Multilabel Counter (PerkinElmer, Inc., Monza, Italy) was used to read the absorbance at 490 nm. Outcomes were presented as percentage survival relative to control cells and expressed as the mean ± standard deviation (SD). Morphological analysis of the samples was carried out by microscopy inspection on an Axio Scope 2 microscope (Zeiss, Oberkochen, Germany).

### 2.5. Hemolysis

Hemolysis assay was carried out using a procedure previously described by Picone et al. [42]. Briefly, 5 mL of venous blood collected from a healthy donor was drawn directly into K2 EDTA coated Vacutainer tubes to avoid coagulation. After centrifugation at 500× *g* for 5 min, the hematocrit and plasma levels were highlighted on the tube. The plasma was then discarded and replaced with 150 mM NaCl, and the tube was centrifuged at 500× *g* for 5 min. This procedure was repeated three times, and in the end, the supernatant was changed with PBS at pH 7.4. An amount of 200 μL of diluted (1:50 in PBS) erythrocytes were layering into a 96-well plate. After that, pieces of bilayer material of different weights (2.5, 5.0, 10.0 mg) or 10 μL of 20% Triton X-100, as a positive control, were mixed with the erythrocytes sample. The plate was incubated at 37 °C for 3 h and then centrifuged for 5 min at 500× *g* to pellet whole erythrocytes. Then, 100 μL of the supernatant was moved from each well into a clear, flat-bottomed 96-well plate and absorbance, due to free hemoglobin presence, was quantified at 490 nm by utilizing a plate reader (Wallac Victor 2 1420 Multilabel Counter (PerkinElmer, Inc., Monza, Italy). After background subtraction, the average absorbance of the positive control was obtained. All experimental data points were normalized with this mean absorbance value, which is the 100% hemolysis. The treated erythrocytes and the control were morphologically investigated by means of a microscopy inspection on an Axio Scope 2 microscope (Zeiss, Oberkochen, Germany).

### 2.6. In Vitro ELISA Assay

Peripheral blood mononuclear cells (PBMc) were isolated from venous blood collected by young and healthy donors and achieved as described by Picone et al. [43]. PBMCs were incubated with lipopolysaccharide (LPS) (0.1 µg mL^−1^) as activators of the inflammatory response, in the presence or not of the bilayer material (10 mg) for 24 h. An enzyme-linked immunosorbent sandwich assay (ELISA) (Thermo Fisher Scientific Inc, Waltham, MA, USA) was performed for quantitative detection of Tumor Necrosis Factor α (TNF-α), Interleukin 6 (IL-6), and Interleukin 1β (IL-1β) in cell culture supernatants.

### 2.7. In Vitro Wound Scratch Assay

Keratinocytes were seeded into 24-well plates at 30 × 10^4^ cells and were maintained at 37 °C and 5% CO_2_ for 24 h to permit cell adhesion and the formation of a confluent monolayer. Subsequently, they were wounded with a sterile plastic pipette tip to leave a scratch of approximately 0.4 mm in width.

The cells were then washed twice with phosphate-buffered saline (PBS), and medium was replaced by a bilayer film extract. All scratch assays were performed in triplicate. Wound closure was monitored, collecting digitized images immediately after scratching, 24 h and 48 h post-induction. Images were analyzed using ImageJ software (NIH). Data has been reported as the extent of wound closure by the initial scratch width.

### 2.8. In Vivo

In vivo experiments were performed in accordance with the guidelines established by the European Communities Council Directive (Directive 2010/63/EU of 22 September 2010) and approved by the National Council on Animal Care of the Italian Ministry of Health; 8–12-week-old male C57BL/6 J mice (Charles River, Calco, Italy) were utilized. All efforts were made to minimize animal pains and suffering and to use the minimal number of animals required to produce reliable results, according to the 3Rs. They were kept under a 12-h light/dark cycle (lights on at 8:00 am), relative humidity of (55 ± 10%), and at a controlled temperature of (21 ± 1 °C).

### 2.9. In Vivo Diabetic Mice Model

Mice were injected with 50 mg/kg, i.p., Streptozotocin (STZ, Merck Life Science, Milan, Italy), dissolved in citrate buffer (sodium citrate, pH 4.5). Animals fasted for four (4) h prior to STZ induction. The STZ-Na Citrate buffer solution was prepared immediately before injection because the drug degrades after 15–20 min in the Na-Citrate buffer. Each mouse was treated with five consecutive intraperitoneal daily injections of STZ. Mice were tested to check levels of hyperglycemia at 4 weeks post-injection by drawing blood from the dorsal vein of the mouse. Animals with blood glucose levels ±250 mg/dL were considered diabetic and used for the experiments.

### 2.10. Cytokines

Skin samples from naive, sham, and bilayer treated animals were collected 2 days, for the healthy mice, and 5 days, for the diabetic mice, post wound induction and snap-frozen in liquid nitrogen (*n* = 5 mice in each experimental group). Cytokines (IL-6, IL-1β, and TNF-α) expression was measured using ELISA quantikine kit (R & D system, Minneapolis, MN, USA), according to the manufacturer’s instructions [44].

### 2.11. Wound Closure

For the evaluation of the wound closure, mice were anesthetized, their dorsal surface was shaved, and a full-thickness excisional wound was induced (diameter 6 mm). A photo of the wound was taken immediately after the biopsy generation (day 0). The bilayer dressing was applied, and pictures at days 0, 3, 5, 7, 9, and 12 were collected and analyzed using ImageJ software to quantify the wound closure rate. The wound closure was calculated as a percentage based on wound size relative to the control group. During the experiments, mice were housed individually and fed with water and food ad libitum. The same procedure was performed for the healthy and diabetic mice.

### 2.12. Histology

After 7 days from wound induction, skin samples were excised and fixed in 10% formalin solution and embedded in paraffin. Serial sections of 5-μm thickness were obtained and stained with Hematoxylin & Eosin (H&E) to evaluate morphology and analyzed with a Leica DM5500 optical microscope (*n* = 5 each group). Migration tongue distance variation was expressed as an absolute distance in μm vs. control group (tip distance was measured with ImageJ software). The results were examined blind.

### 2.13. Statistical Analysis

For the statistical analysis of the proposed experiments, ANOVA was utilized to evaluate statistical significance, followed by Bonferroni’s post-hoc test. GraphPad Prism 5 was utilized for all statistical analysis (GraphPad Software Inc., San Diego, CA, USA). Results with a *p*-value *<* 0.05 were considered statistically significant.

## 3. Results and Discussion

### 3.1. In Vitro

#### 3.1.1. Biocompatibility and Hemocompatibility

**Figure 1:**
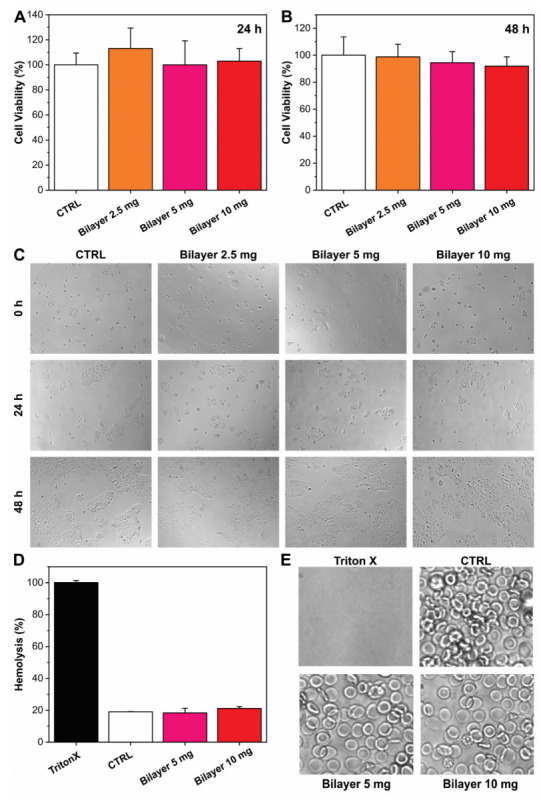
(**A**,**B**) Viability of HaCaT cells after 24 h and 48 h exposure to bilayer extraction media. Values were normalized to control (set to 100%). Average percentage values ± S.D. of three independent experiments. (**C**) Photos of keratinocytes in the CTRL and bilayer samples at different time points. (**D**) Histogram relative to the absorbance of released Hb after treatment with 4 and 6 mg of bilayer films. The values are expressed as % with respect to the positive control (TritonX). (**E**) Optical images of erythrocytes after the treatment with Triton X, medium, 5 mg, and 10 mg of bilayer, respectively.

The cytotoxicity effect of the bilayer films was firstly studied in the HaCaT cell line, and the results are reported in Figure 1A–C. The extracts of bilayer films did not affect the viability of HaCaT cells both after 24 and 48 h of exposition. Therefore, according to ISO10993-5 guidelines, where the limit of toxicity is defined as a reduction of 30% of the viability, the bilayer resulted as biocompatible. The morphological analysis of the cells confirmed what was observed with the CellTiter-Glo assay, and no alterations in the cell shape were noticed. Similar outcomes were obtained when the bilayer extract was tested on the HDFa cell line, and the main results are shown in Figure S1A,B. Additionally, in this case, the bilayer resulted in being fully biocompatible along all the concentrations under investigation.

The application of materials in fresh and open wounds require biocompatibility and inertia, not only with respect to cells that compose the tissue (fibroblasts and keratinocytes), but also with respect to the corporal fluids [43]. Therefore, red blood cells were tested in the presence of the bilayer. Specifically, blood was incubated with bilayers of different weights (5 and 10 mg) for 3 h, and the hemolysis ratio was analyzed by measuring the amount of free plasma hemoglobin (Hb). As shown in Figure 1D, the hemolysis percentage induced by the presence of the bilayer in the blood sample was comparable to the untreated control, whereas total hemolysis was observable in the Triton-X control sample. The result was also confirmed by erythrocytes microscopic observation (Figure 1E), suggesting the excellent hemocompatibility of the material.

#### 3.1.2. Anti-Inflammatory Assay

**Figure 2:**
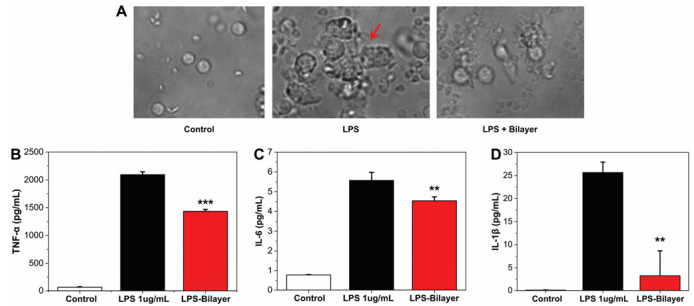
(**A**) Optical images of control samples of PBMC cells; PBMCs and LPS; and PBMC, LPS, and bilayer, respectively. The red arrow indicates the clusters caused by the LPS presence. (**B**–**D**) Histogram of the level of TNF-α, IL-6, and IL-1β quantified by ELISA and expressed in the control samples of PBMC cells; PBMCs and LPS; and PBMC, LPS, and bilayer. ** *p* < 0.01 vs. the LPS treated group; *** *p* < 0.001 vs. the LPS treated group.

The inflammatory response was measured as production of three inflammation mediators TNF-α, IL-6, and IL-1β. PBMCs were stimulated by LPS in the presence or not of the bilayer, and microscopic images of the three systems are reported in Figure 2. As can be noticed, LPS activation caused the formation of cell clusters [43]. Instead, this toxic effect is morphological inhibited in the samples with the bilayer. In agreement, the result obtained by ELISA test indicated that, after LPS stimulation, an increasing of the release of TNF-α, IL-6, and IL-1β by the PBMCs was observed (Figure 2B–D). On the contrary, when the bilayer construct was present, a significantly reduction of cytokines levels was detected (Figure 2B–D), indicating that the material has anti-inflammatory properties.

#### 3.1.3. In Vitro Wound Scratch

**Figure 3:**
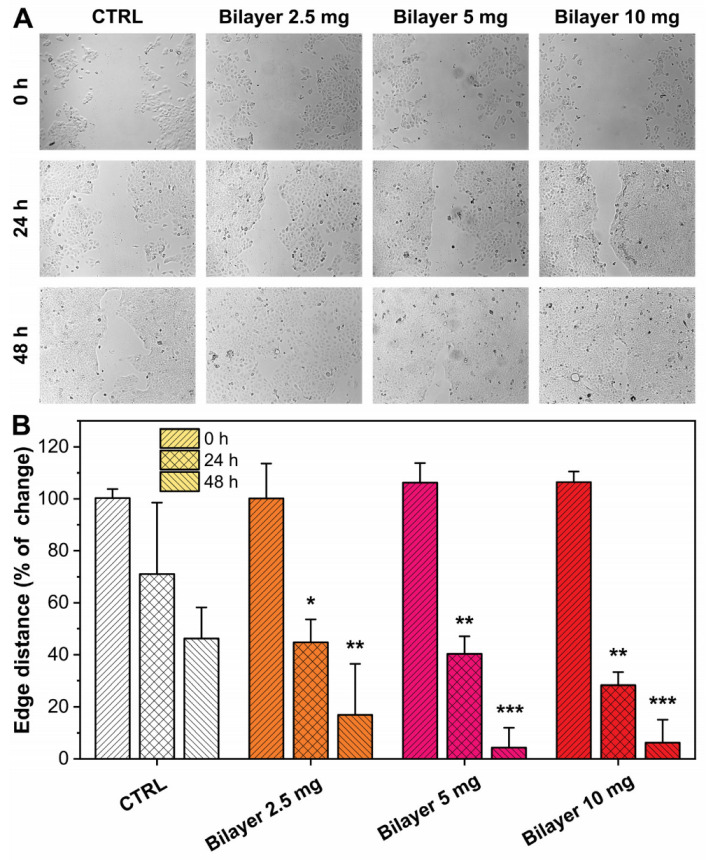
(**A**,**B**) Images and results, respectively, of migration effects on HaCat cells in the in vitro wound scratch assay observed at 24 h and 48 h for the bilayer at different concentrations (mg/mL). Wound closure was calculated using Image J software. Results are expressed as Wound closure rate %. Three different areas were measured in each well. * *p* < 0.05 vs. the control group; ** *p* < 0.01 vs. the control group; *** *p* < 0.001 vs. the control group.

HaCaT cells were grown to confluence and scratched with a 10 µL sterile pipette tip. Detached cells and debris were washed away with PBS. Cells were incubated with or without bilayer film extract at different concentrations and observed for 24 and 48 h post-wounding to assess the effect on wound closure rate. Fresh medium supplemented with serum was used as a control. Distance between the edges of the wound monolayer was measured by ImageJ software as the mean distance. The results are reported in Figure 3. Images of the different conditions at 0, 24, and 48 h are displayed in Figure 3A. The derived outcomes highlighted that, after 24 h, the keratinocytes treated with different concentrations of bilayer were capable of faster healing of the wound scratch with respect to the untreated sample. Specifically, after 24 h, the wound closure in the control samples was less than 35%, whereas it had already reached 69–73% in the presence of the bilayer samples. At 48 h, the wound was almost recovered in the presence of the bilayer (87–95%), while CTRL was less than 65%. These observations suggest the capacity of this formulation to positively affect the proliferation of keratinocytes.

### 3.2. In Vivo

#### 3.2.1. Anti-Inflammatory Properties

**Figure 4:**
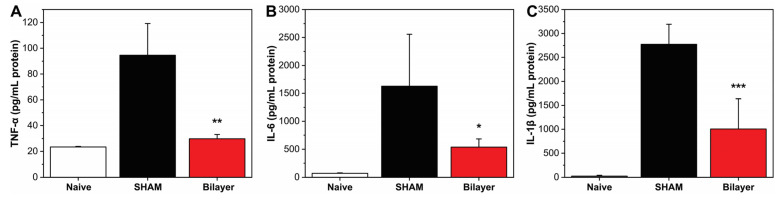
In vivo evaluation of TNF-α (**A**), IL-6 (**B**), and IL-1β (**C**) after 48 h from wound induction. * *p* < 0.05 vs. the wound untreated group; ** *p* < 0.01 vs. the wound untreated group; *** *p* < 0.001 vs. the wound untreated group.

Inflammatory cytokines such as TNF-α, IL-6, and IL-1β are released at the wound site during the inflammatory phase, exerting an important action in the healing process: they stimulate the proliferation and chemotaxis of fibroblasts and increase the production of extracellular matrix proteins [45,46]. These factors have been shown to be fundamental in their action, even in wounds aggravated by diabetes, for instance. Cutaneous levels of TNF-α, IL-6, and IL-1β were analyzed in mice treated with the bilayer and untreated (SHAM) after 48 h from the wound induction. In addition, a naive group was also taken into consideration during the experiments. As expected, naive animals expressed a low level of both cytokines, confirming that no inflammation was present. On the contrary, a significant increase of TNF-α, IL-6, and IL-1β was observed in wounded/sham animals. Instead, in the mice treated with the bilayer dressing, a reduction of ≈69, 67, and 64% for TNF-α, IL-6, and IL-1β, respectively, compared to the wounded/SHAM levels was observed, Figure 4A–C. These results highlighted and confirmed an important role of the bilayer in the modulation and reduction of the inflammatory response, extending its potential application in cases where the level of these inflammation mediators is enhanced, such as burns, diabetic ulcers, and chronic wounds.

#### 3.2.2. Evaluation of the Wound Healing Rate

**Figure 5:**
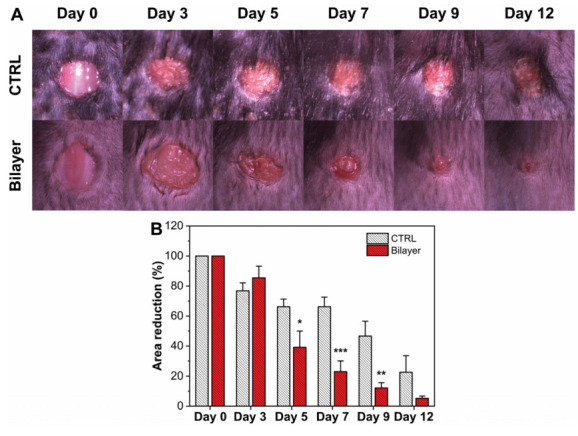
(**A**) Representative photographs of the skin of wound-healing mice at different time points (day 0, day 3, day 7, day 9, and day 12): untreated (no dressing) and bilayer dressing treated wounds are shown. (**B**) Time-course (days) of wound healing in mice untreated (white bars) and treated with the bilayer (red bars). * *p* < 0.05, ** *p <* 0.01, and *** *p* < 0.001, compared to untreated mice.

The capacity of the bilayer constructs to accelerate the healing process was investigated in a full-thickness excisional wound-healing mice model, and the main results are reported in Figure 5A,B. As can be noticed in the graph of Figure 5B, bilayer materials were able to reduce statistically (*p* < 0.05) the wound area with respect to the control starting from day 5. Furthermore, the wound closure was obtained in only 12 days for mice treated with the bilayer, suggesting faster and more effective healing in these animals.

#### 3.2.3. Anti-Inflammatory Properties in Diabetic Mice

**Figure 6:**
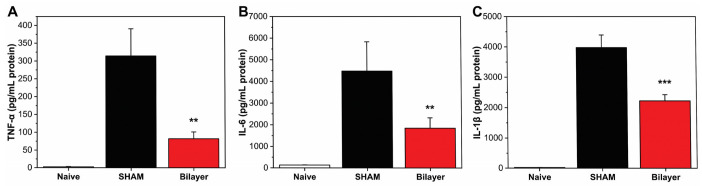
In vivo evaluation of IL-6 (**A**), IL-1β (**B**), and TNF-α (**C**) levels in diabetic mice after 96 h from wound induction. ** *p <* 0.01, and *** *p* < 0.001, compared to untreated mice.

Expression levels of IL6, IL-1β, and TNF-α were analyzed after 96 h from wound induction because, in the diabetic mouse model, the inflammatory process is delayed, as shown in the healing process [46]. A significant increase of TNF-α, IL6, and IL-1β was shown in SHAM animals. Such increased levels of cytokines expression, instead, were strongly reduced in mice treated with the bilayer dressing, as can be noticed in Figure 6A–C. Specifically, a reduction of ≈74, 59, and 44% for TNF-α, IL-6, and IL-1β, respectively, was observed. These data confirm that the developed bilayer films are also active in the diabetic mouse model, where the injury is more severe than in normal mice. Indeed, impairment of the inflammatory response is one of the reasons for the stopped healing process in diabetic ulcers and complicated wounds. For instance, pro-inflammatory cytokines such as TNF-α, IL-6, and IL-1β are released by neutrophils and activated macrophages, leading to increased matrix metalloproteinases (MMPs) production and reduced tissue inhibitor MMPs, blocking the ECM formation and the proliferation process [9,47]. Therefore, reducing these cytokines is a key point in the potential treatment of these chronic wounds.

#### 3.2.4. Wound Healing in Diabetic Mice

**Figure 7:**
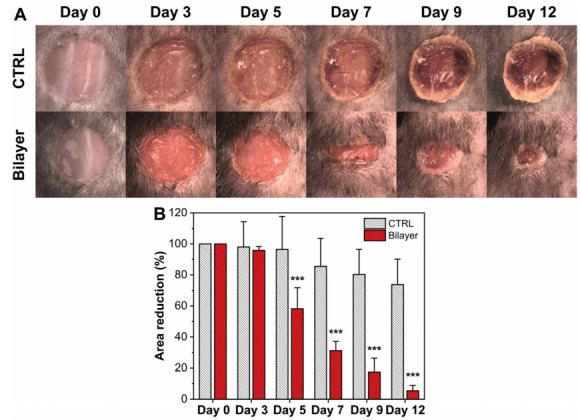
(**A**) Representative photographs of the skin of diabetic mice wound healing at different time points (day 0, day 3, day 5, day 7, day 9, and day 12): untreated (no dressing) and bilayer dressing treated wounds are shown. (**B**) Time-course (days) of wound healing in mice untreated (white bars) and treated with the bilayer (red bars). *** *p* <0.001, compared to untreated mice.

In diabetic subjects, the healing process of injury is normally delayed compared to normal people; for this reason, the reduction of wound area was evaluated in a diabetic mouse model to verify the activity of our material. As can be noticed in Figure 7A, the wound healing in the STZ mouse model was markedly delayed compared to the control healthy mice (Figure 5A). After 5 days, the presence of the bilayer significantly enhanced the healing rate with respect to the untreated diabetic mice. After 12 days, the diabetic mice treated with the bilayer could solve the injury and demonstrate an efficient healing, while in the untreated mice, the wound remained blocked at the levels of the early stages.

**Figure 8:**
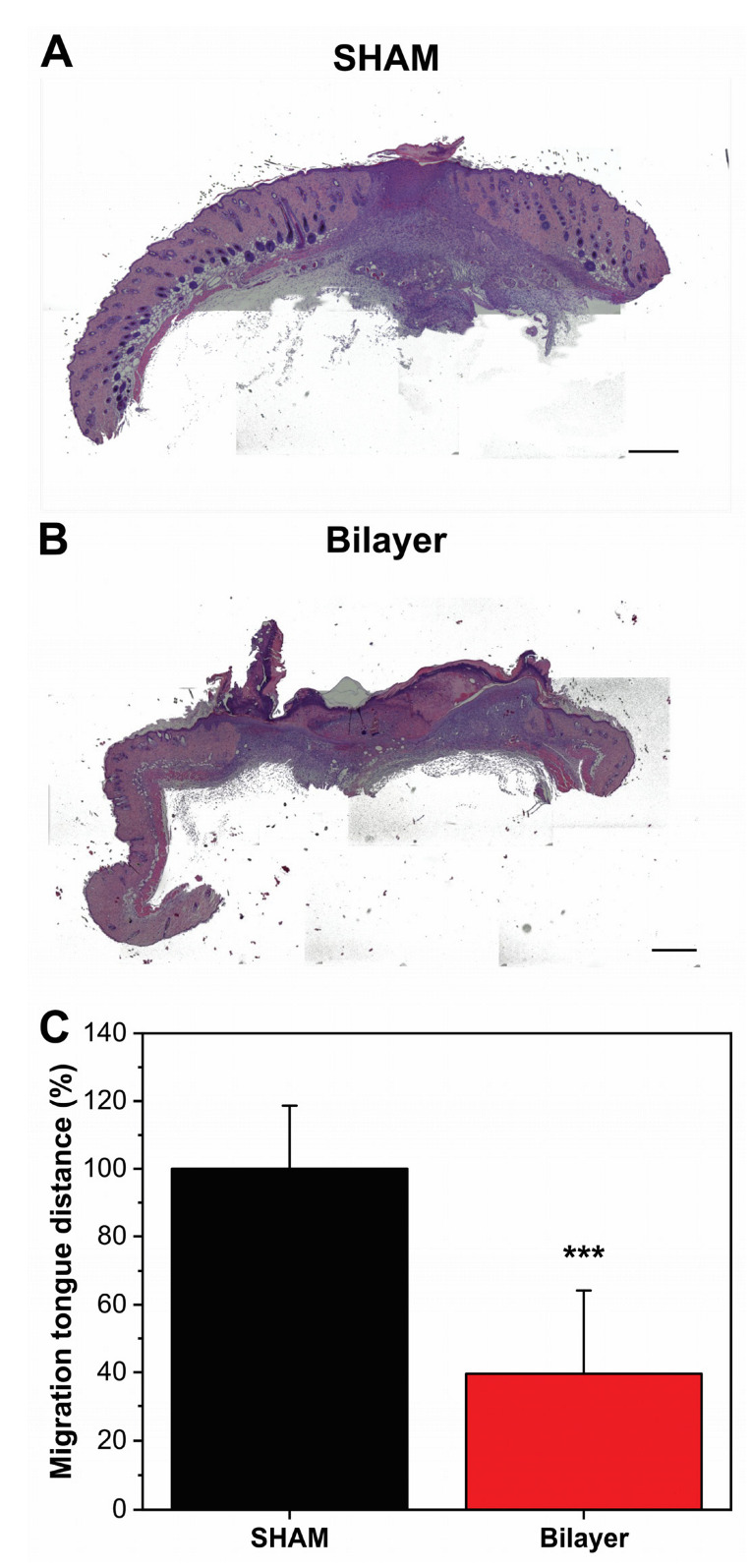
(**A**,**B**) Hematoxylin-eosin staining of skin sections from diabetic untreated and treated mice (scale bars, 200 µm) 7 days after wound induction. (**C**) Migrating tongues distances percentage in diabetic untreated and treated mice in wound closure evaluation in full-thickness excisional skin wounds model. *** *p* < 0.001 vs. the wound untreated group.

Furthermore, histological analysis was carried out to confirm the observation of the wound closure experiments. Hematoxylin and eosin stained dorsal skin sections from untreated and treated diabetic mice are reported in Figure 8A,B, respectively. For evaluating the healing process state, the distance between the tips of migrating epithelial tongues of wound bed region in the two different groups was measured as pixel distance with ImageJ software (Figure 8C). Results showed that, in bilayer treated animals, the migration tongue distance was significantly reduced (by ≈60%) compared to the control one. These outcomes suggest the capacity of the bilayer material to be active also in the case of diabetic wounds.

## 4. Conclusions

In this work, we evaluated a PVP/hyaluronic acid-based bilayer material in different in vitro and in vivo models. The dressing resulted in being fully biocompatible when tested on fibroblasts and keratinocytes cell lines, and no hemolytic effects were observed after contact with red blood cells. Enhancement in the proliferation of keratinocytes in the presence of bilayer was demonstrated in an in vitro wound scratch model. The release of pro-inflammatory mediators from peripheral blood mononuclear cells in the presence of a positive stimulus such as LPS was significantly reduced when the bilayer film was also present. The levels of TNF-α, IL-6, and IL-1β resulted in decreases, both in healthy and diabetic mouse skin when wounds were covered with the bilayer. Finally, the wound healing capacity of the bilayer dressing was demonstrated. The material accelerated the wound closure, both in healthy and diabetic mice. In addition, histological analysis confirmed the capability of the bilayer for proper skin regeneration in the diabetic wound model.

Combined with easy and scalable fabrication and adhesive and antibacterial properties described in our previous work [41], these outcomes demonstrate how the presented multifunctional bilayer dressing can be a suitable tool for the treatment and management of a wide variety of skin damages, from acute to chronic.

## Supplementary Materials

The following are available online at https://www.mdpi.com/article/10.3390/pharmaceutics14030483/s1, Figure S1: (A,B) Histogram and images of fibroblasts viability after 48 h of untreated and treated with the bilayer material at different quantities (2.5, 5, 10 mg).
