# Supplementary material for: Evaluation of a Multifunctional Polyvinylpyrrolidone/Hyaluronic Acid-Based Bilayer Film Patch with Anti-Inflammatory Properties as an Enhancer of the Wound Healing Process
Source: Pharmaceutics. 2022 Feb 22;14(3):483. doi: 10.3390/pharmaceutics14030483 (PMC8955039; doi:10.3390/pharmaceutics14030483)
Supplement: Supplementary file 1 [file pharmaceutics-14-00483-s001.zip › pharmaceutics-1594496-supplementary.pdf]

pharmaceutics

Supplementary Materials: Evaluation of a multifunctional polyvinylpyrrolidone/hyaluronic acid-based bilayer film patch with anti-inflammatory properties as an enhancer of the wound healing process

Marco Contardi, Maria Summa, Pasquale Piconec, Ornella Roberta Brancatoc, Marta Di Carloc, Rosalia Bertorellib, Athanassia Athanassioua

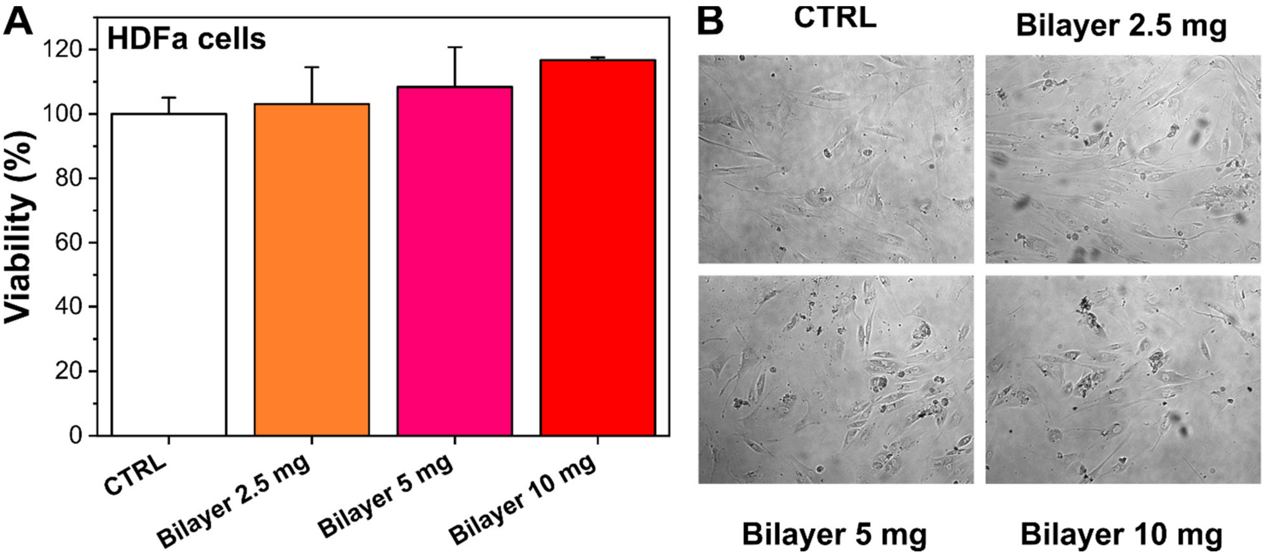

Figure S1. (A,B) Histogram and images of fibroblasts viability after 48 hours of untreated and treated with the bilayer material at different quantities (2.5, 5, 10 mg).
